# Navigating their child’s attachment-related difficulties: Parents’ journey from shame into awareness

**DOI:** 10.1177/13591045221135993

**Published:** 2022-11-09

**Authors:** Chloe Crompton, Ming W Wan, Sarita Dewan, Anja Wittkowski

**Affiliations:** 1 School of Health Sciences, The University of Manchester, Manchester, UK; 2 Greater Manchester Mental Health NHS Foundation Trust, Manchester, UK; 3 Manchester Academic Health Science Centre, Manchester, UK; 4 Pennine Care NHS Foundation Trust, UK

**Keywords:** Insecure attachment, attachment disorder, clinical formulation, child and adolescent mental health service, mental health services, grounded theory, shame

## Abstract

Attachment-related difficulties frequently present in child and adolescent clinical services. Yet how parents engage with being informed of their child’s attachment-related difficulties is little understood. In this qualitative study, ten parents with a birth child with attachment-related difficulties, as informed by a relevant service, and six healthcare professionals, were interviewed. The aim was to explore both perspectives on how parents experienced and engaged with this process, of their understanding of the child’s difficulties and the supports they engaged with. Using grounded theory, the parental journey from shame to awareness is described, based around four main themes: *failing as a parent*, *the process of making sense*, *a call to action,* and *awareness of attachment and interrelated difficulties*. The intensity of shame and defensive processes felt by parents came through strongly in narratives, forming a key barrier to sense-making and action, while specific clinical, personal and support/resource characteristics facilitated progress. The findings highlight how parents can be better supported into a space of attachment-related awareness and understanding, which may in turn facilitate more positive outcomes for the child. The study also raises wide ranging implications relevant to all involved in the investment, planning and delivery of care for this client group.

## Introduction

Relational attachment processes have become an integral part of understanding child mental health because young children’s mental health and any subsequent interventions are affected by their relationships, especially with their parents. ‘Attachment difficulties’ is often used as an umbrella term that refers to significant relational difficulties on the continuum between childhood insecure attachment patterns (which are common, non-diagnostic and relationship-specific) and attachment disorders (which are rare, diagnoseable, and pervasive across all relationships) (National Institute of Care and Excellence, 2015). This term may be used by healthcare professionals (HCPs) in child clinical mental health services to describe how the child’s significant difficulties have developed or are being maintained, as derived from the professional’s assessment and psychological formulation of the family, particularly when the child’s presenting problems are associated with the parent-child relationship without representing a diagnosable attachment disorder.

To our knowledge, there has been no previous study of the lived experiences of biological parents of a child considered to have attmacheninformation t difficulties, nor of HCP perspectives of this parental experience. Receiving clinical that one’s own child has ‘attachment-related difficulties’ can be highly emotive for parents since attachment difficulties are understood to be largely shaped by how caregivers respond to their child (Bowlby, 1969; 1988; Koehn & Kerns, 2018). Understanding this experience is clinically informative since it is likely to affect parents’ subsequent engagement with the information and intervention work and in turn impact on child outcomes. Furthermore, parenting a child with attachment difficulties is highly demanding, and parents may welcome clinical recognition and new understanding of the issue, similar to the relief reported by parents of children with newly diagnosed neurodevelopmental conditions (Braiden et al., 2010; Smith-Young et al., 2020).

The limited research on parents’ experiences of their children’s attachment difficulties has focused on adopted children. Such studies reported that their child’s attachment-related disruptive behaviour destabilised the parents’ confidence in parenting (Follan & McNamara, 2014), and led to a barrage of negative feelings, including feeling judged, unsupported and unprepared (Beek, 1999), and relational strain in the wider family (Baumgart, 2020). Studies of parental responses to receiving a diagnosis for childhood disorders, such as autism or a chronic health condition, also commonly report strong emotional reactions, including disbelief, guilt and responsibility (Ashtiani et al., 2014; Fletcher, 2016; Smith-Young et al., 2020). While challenges remain, parents later describe psychological growth and change in perspective (Waizbard-Bartov et al., 2019).

Driven by the intention to develop a shared understanding, this qualitative study explored the perspectives of UK parents and HCPs to understand (a) the experiences of biological parents following being informed by a professional that their child aged 3–16 years had attachment-related difficulties, (b) how they made sense of this information and (c) what factors affected their engagement with support.

## Method

### Design

The study utilised constructivist grounded theory (Charmaz, 2014) to understand parents’ experiences based on semi-structured telephone interviews. As an under-theorised topic, grounded theory methodology was employed to support theory development around parental experiences ‘grounded’ in the data. Experts by experience were consulted during the design process. Ethical and other relevant approvals were granted (ref: 20/NW/0019).

### Participants

Between July 2020 and February 2021, parents were recruited through social media advertising and HCPs via participating National Health Service Trusts in northwest England. Eligible participants were: (1) English-speaking parents >18 years of age who had been informed that their birth child (aged 3–16 years) had attachment-related difficulties or received an attachment-related formulation (henceforth both will be referred to as attachment-related ‘formulation’) from a Child and Adolescent Mental Health Service (CAMHS) or related professional service (i.e. health or education) in the last 3 years; (2) fully qualified HCPs who had delivered at least one attachment-related formulation to a biological parent and had at least 4 months’ CAHMS work experience. Parents with significant mental health difficulties or whose child had been removed from their care were ineligible.

### Interviews

An initial interview topic guide was developed informed by a literature review, piloted and refined. The first participants were recruited, interviewed and analysed in turn, consistent with grounded theory methodology. Analysis explicitly shaped the direction of further interviews, enhancing theory construction based directly on reported experience (Dey, 1999). As prominent categories emerged, further participants were recruited aligned with theoretical sampling (Glaser & Strauss, 1967), until theoretical sufficiency was reached (i.e., satisfactory data to develop categories into an integrative theory).

Parent interviews explored their understanding of the formulation and how they made sense of this information, while HCP interviews explored their experiences of delivering these formulations (for copies see Appendix A and Appendix B).

### Data analysis

Grounded theory involves a process of obtaining and analysing data using a constant comparison of all parts of the data with one another, to develop a theory directly based on the reported experience of participants (Strauss & Corbin, 1997). Thus, grounded theory was chosen because we were most interested in developing an understanding (or theory) shared and derived from parents as well as HCPs. The analysis of initial data explicitly shaped the direction of further data collected, permitting concepts to be explored in more depth and enhance the construction of the theory (Dey, 1999). The use of this constant comparative method allowed the data to be checked within and between interviews and it allowed for the examination of similarities and differences so that codes, categories and concepts were consistent and representative of the data. The premise of theoretical sufficiency (Dey, 1999) was used as the guiding principle for data collection rather than theoretical saturation (Charmaz, 2014), whereby we aimed to collect satisfactory data to develop categories into an integrative theory.

Repeatedly reading interview transcripts and daily listening to the interviews over seven days enabled the main researcher (CC) to become immersed in the data to help provide theoretical insight (Corbin & Strauss, 2008). After line-by-line coding (assigning a concise code that captured meaning for that data segment) and focussed coding (grouping similar codes to form carefully defined categories), categories were organised into overarching themes. Data checked within and between interviews ensured that codes, categories and concepts were consistent and representative of the data (constant comparative method). Theoretical coding then took place which involved finding the core concepts that linked the codes and categories identified during initial and focussed coding. The codes were refined by linking them to each other and to the existing literature until the theory began to emerge (Charmaz, 2004). Throughout, memo-writing and diagramming captured possible connections and insights to facilitate this theory development (Kennedy-Lewis, 2014). Quality checks included the independent double coding of the first half of four transcripts (discrepancies discussed and resolved) and the sharing of the developed theory with three study participants to check that it resonated with their experience.

### Rigour and credibility

Open-ended interview questions were used to to minimise interviewer influence and maintain neutrality (Glaser & Strauss, 1967). Verbal feedback did not approve, disapprove or confirm perspectives. The main researcher (a parent as well as a HCP with CAMHS clinical experience and a keen interest and some training in attachment theory and concepts) had limited experience of delivering attachment-related formulations, which allowed the analysis to be conducted from a non-expert standpoint. To address any subjectivity issues and ensure the analysis process was rigorous, the emerging concepts were regularly discussed within the team and a journal was used to enable reflection.

To ensure credibility, the first half of four transcripts were double coded by independent coders. Any discrepancies were discussed and resolved. The developed theory was visually presented and shared with three participants to find out if it resonated with their experience which afforded further credibility and validity to the study and its derived theory.

## Results

Of the 23 participants assessed for eligibility, 6 parents were ineligible (not a biological parent: *n* = 3; not informed of child attachment difficulties: *n* = 3) and 1 withdrew before the interview due to personal circumstances. The final sample comprised 10 parents and six HCPs (see Table 1). All parents discussed one child, except one who discussed two of their children. One parent was specifically referred for ‘diagnoses’ and treatment of attachment difficulties, whilst the remainder received a clinical formulation following a period of assessment for mental health or behavioural difficulties. None of the parents or HCPs in this study were involved in child protective services or social care. Parent participants reported their childrens’ attachment-related difficulties predominantly presenting as extreme difficulties with emotion regulation, significant distress with separation from primary caregiver and difficulties trusting others.

**Table 1.** Participant characteristics.

*Parent participants (n=10)*	*n*	*HCP participants (n=6)*	*n*
Participant parent		Job title	
Mother	8	Clinical psychologist	3
Father	2	Mental health practitioner	2
Age (years)		Psychiatrist	1
Under 30	1	Age (years)	
31–40	3	Under 30	2
40+	6	31–40	4
Gender		Gender	
Female	8	Female	5
Male	2	Male	1
Ethnicity		Ethnicity	
White British	8	White British	4
Other	2	Other	2
Marital status		Years professional experience	
Single	1	0–5	2
Married	6	6–10	2
Cohabiting	3	11–15	2
Employment status			
Employed	7		
Other	3		
Child being discussed			
Daughter	2		
Son	9		
Other children			
Yes	9		
No	1		
Child has other conditions/syndromes/difficulties			
Yes	9		
No	1		
Child age when informed of attachment difficulties (years)			
6	3		
7	2		
8	3		
9	3		
Current age of child (years)			
Under 10	6		
11+	5		

Audiotaped interview durations ranged 34–99 minutes. Sixteen hours, 43 minutes and 40 seconds of audio data were transcribed.

### The overarching model

**Figure 1.**
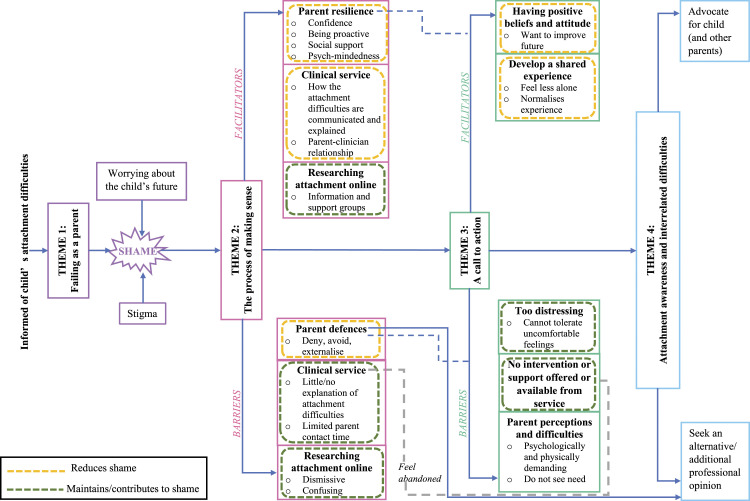
Grounded theory of “parents’ journey from shame into awareness”.

The diagrammatic model in Figure 1 presents four superordinate themes with their associated subthemes and the relationships between them that arose in the narratives. The grounded theory depicts the journey parents made from shame to awareness following being informed of their child’s attachment-related difficulties (for a representation of the full development of the grounded theory, see Appendix C). Parents’ awareness here refers to that of accepting and having a contextualised understanding of their child’s attachment and of the parent’s role. Although participants discussed their experience as a journey, it was not always linear and was strongly impacted by shame, the core concept in participant narratives. Those parents who presented a better state of awareness appeared to experience less shame.

From feeling like they had failed as a parent, the journey to awareness depended on how they made sense of the information and engaged with support –with facilitators and barriers at each stage. For example, in making sense of the information, the journey to awareness of those parents seeking a different diagnosis halted because they moved into a journey of understanding and making sense of a different diagnosis. At the model’s endpoint, parents evidenced a capacity to reflect and mentalise about the child’s issues. Many parents recognised the attachment difficulties as part of a larger picture with interrelated difficulties, yet their sense of shame did not resolve entirely.

The following summarises the superordinate and sub-themes (see Table 2 for more sample quotes).

**Table 2.** Themes and supporting quotes from parents and HCPs.

Superordinate theme and subordinate themes	Sample quote
1. Failing as a parent	
1.1. Guilt and self-blame	*“I was questioning all the things I’d done over the years and how I could have changed things (…) You feel like it’s your fault”* (parent2:272–275)
1.2. Stigma	*“…it’s just very different and people look at you weird. There’s so many people out there who have no idea, and lucky them they haven’t had to (…) deal with those difficulties. It feels like they look at you and question your parenting” (parent2:197-199)*
1.3. Worrying about the child’s future	*“It’s not like when you have a child that’s got a little bit poorly, you know, that child’s going to get better. This is a lifelong thing”* (parent7:559–561)
2. The process of making sense	
2.1. Facilitators to the sense-making process	*“He always drew things like flow charts and stuff on a white board for me and said (…) so he’s making sense of the behaviour for (name anonymised) so I could understand that (name anonymised) with the way he was, for the reason he was, that helped me so much (…) that I understood it wasn’t coming directly from something I had done” (parent4:360-363)*
2.2. Barriers in the sense-making process	*“…but if you tell me it’s ADHD I can give him a tablet a day and things get easier and better and I will so much more prefer that, than all the stuff you’re going to have me dig deep for”* (HCP1:357–359) *“…but then he said ‘there’s definitely some attachment issues’ and I was just like ‘OK, so how?’ and then he didn’t elaborate. He said it’s too early and will take some time to unpick. And that was it! He never gave me any (…) explanation to why he believed it was attachment. I felt like a floating kite”* (parent6:315–318)
3. A call to action	
3.1. Facilitators to accessing and engaging with support/intervention	*“…maybe in a month or two we’ll have answers and I’ll be able to sort of move on a bit more and start accessing support for (name anonymised) and our life will be better in a couple of months time”* (parent1:272–275)
3.2. Barriers to accessing and engaging with support/intervention	*“…it was like a spark to a bloody powder keg! It was awful. It made him worse, awful it was. All about me separating from (child) in order for their work to start, was awful and (child) found it highly distressing”* (parent5:397–400) *“Whereas with attachment difficulties, I guess it’s so much more about making changes yourself for your family, which is really tough when you’re maybe feeling rubbish or, erm, you know, you’re struggling so much anyway, to then try and dig deep and then even deeper to those internal resources (…) erm so in that sense it must be really hard”* (HCP1:365–367)
4. Attachment awareness and interrelated difficulties	
4.1. Advocating for my child (and other parents)	*“It’s still an ongoing battle. It’s still an ongoing thing where we’re trying to improve life for him and the family. We’re still fighting the system to try and get the right support for him. And (…) fighting against the system to get them to help us to understand exactly what’s going on for (child), I suppose the more we understand what’s going on for him, the more we can help him and the more we know why he does what he does”* (parent5:443–447)
4.2. Seeking an additional/alternative professional opinion	*“So, when he got his ADHD diagnosis, I cried. It was like someone saying ‘I get it, I understand, I agree, you’re not losing your mind…”* (parent6:192–195)

### Superordinate Theme 1: Failing as a parent

All the parents experienced a sense of failure after being informed that their child had attachment-related difficulties. Parents experienced self-blame and guilt, felt a sense of stigma and worried about their child’s future.

### Subordinate Theme 1.1: Guilt and self-blame

Parents internalised a lot of guilt and questioned their parenting abilities: “*did I do something wrong or where did I go wrong with bringing him up”* (parent3:75–77). Some parents used sensory metaphors to convey the immediate physiological impact of being informed about their child’s attachment-related difficulties: “*visceral body blow*” (parent5:131), “*kicked in the stomach*” (parent4:221);*“I remember feeling like I was underwater and like I couldn’t even feel the air around me, it felt like sounds were really far away and I felt like I was floating. It was so many strange sensations”* (parent5:282–284). These strong negative feelings led to withdrawal, low self-esteem and mood, intrusive thoughts, weight gain and self-harming.

### Subordinate Theme 1.2: Stigma

Attachment difficulties were highlighted as stigmatising as the concept is not normalised In society. HCPs spoke about how attachment is widely considered to be “*synonymous with abuse*” (HCP1:245) and has strong associations with trauma. On the other hand, parents discussed feeling stigmatised because the lack of opportunities to talk about attachment contributes to poor societal awareness which further reinforces the lack of wider conversations. This leaves parents feeling isolated and estranged from their family and peers.

### Subordinate Theme 1.3: Worrying about the child’s future

Participants spoke about how, universally, parents hold expectations about their child’s future. Upon being informed about their child having attachment-related difficulties, parents worried that their child’s future looked complex because the label of attachment was perceived by some as lifelong. Parents felt hopeless and believed that they were the cause of their child’s lost future.

### Superordinate Theme 2: The process of making sense

Parents were intrinsically motivated to engage in the process of making sense of their child’s attachment formulation to seek comprehensibility and meaning because of their strong emotional reaction and lack of initial understanding of attachment. It involved appraising how attachment difficulties explained their child’s behaviour through considering their child’s early relational experiences.

### Subordinate Theme 2.1: Facilitators to the sense-making process

Participants discussed the parents’ proactive seeking of information to enhance understanding, hope and connection. Parents described buying books, reading literature, attending attachment-based training, liaising with professional bodies, accessing therapy or counselling, and joining local support or online community groups related to attachment difficulties and disorders. The latter was particularly helpful for quick advice from people who had been in similar situations to them and for feeling less isolated. Online searches could be helpful for enhancing understanding, though HCPs added that this would be the case if parents were psychologically minded so they could better discern relevant information.

Positive HCP characteristics and communication also facilitated sense-making. Parents valued HCPs who were kind, relatable, a good listener and non-judgemental. These helped parents to feel safe, and were perceived to provide trustworthy information. Receiving a contextualised attachment formulation in a timely, non-judgemental and sensitive way was important to parents. HCPs talked about how parents found it helpful when they worked collaboratively to *“make links along the way”* (HCP1:181), shared their meanings of attachment, contextualised their hypothesis and placed emphasis on how the parent had ‘done enough’. Such communications validated and normalised parents’ experiences and helped them to *“mentalise”* (HCP4:121), placing them in a better position to develop more accurate meanings of the formulation and attachment.

### Subordinate 2.2: Barriers to the sense-making process

HCPs described parents’ strong avoidance or denial of their child’s attachment difficulties. The information was too difficult for them to accept due to associated strong feelings of being blamed and led some to seek a neurodevelopmental diagnosis (predominantly autism spectrum conditions or attention deficit hyperactivity disorder). HCPs interpreted this parental reaction as lessening the parents’ sense of personal responsibility in potentially having to face and relate their own attachment patterns and histories. From the HCP perspective, an attachment formulation can be easy for parents to dismiss if they need to defend against their own significant adverse life events and how these might impact their child’s life.

Online information describing the impact of an attachment difficulty or disorder looked “*alarming*” (HCP6:317 & HCP3:118) to parents, eliciting shame and concern. HCPs perceived that parents consequently drew their own conclusions and generated their own hypotheses of the situation, denying or dismissing information if they felt criticised or distressed. Such defences were notably absent from parent narratives. They reported confusion by the numerous terms, diagnoses and types of attachment that they encountered from online searches which often offered more questions than explanations. Some parents recalled being provided with little explanation or evidence when they received the attachment-related formulation, which left them directionless and ashamed. However, HCPs did consistently refer to services as under-funded, under-resourced, with a lack of a consistent approach across services for receiving or managing referrals or attachment-related cases. HCPs reported feeling anxious about the available interventions for these families, which were time-limited and lacked the necessary depth to affect real change and may influence the quality of the formulation they provided.

### Superordinate 3: A call to action

Parents accessed a range of services; five participated in attachment-related interventions, four in parenting interventions, four had individual psychological support for their child through their local child mental health service, and three had psychotherapy or counselling (self-funded) due to the shame they experienced.

### Subordinate 3.1: Facilitators to accessing and engaging with support/intervention

Participants discussed how parents accessed support to understand their child better, to “*reach*” (parent5:414) their child and improve their child’s prospects. Such positive, motivated engagement developed their understanding of their child and themselves. Parents also discussed how the feeling of a sense of community and shared experience within support groups helped normalise parental experience resulting in them feeling less alone, validated, and less stigmatised. Engaging with group support in particular allowed parents to interact and achieve connection with others, allowing them to develop insight and build on their capacity to reflect about themselves and their child in the context of other people’s stories, and reduced their sense of self-blame and shame.

### Subordinate 3.2: Barriers to accessing and engaging with support/intervention

Parents highlighted that, generally, clinical services offered no aftercare once informed of their child’s attachment difficulties, leaving parents to feel rejected or abandoned by the service that was supposed to help them: *“it felt like our plight was irrelevant”* (parent8:329). HCP reports support parental narratives in that parents were often told about their child’s attachment difficulties, with little follow-up support, which they attributed to a lack of evidence-based interventions for biological families and service under-funding.

Where intervention was offered, HCPs described ‘defensive’ parents as less likely to engage because they did not see a need for intervention work and had not appraised issues as attachment-related. The interventions offered were perceived as likely to be too psychologically demanding if parents may be going through their own mental health difficulties, or required resources parents lacked (e.g., time, transport, finances and childcare). Therefore, attachment-related support was not viewed as a priority by HCPs. Some parents reported that participating in the intervention they were offered was distressing, especially parents whose child had accessed psychological intervention. Some stopped engaging because they could not tolerate the uncomfortable feelings it generated. These parents’ emotional experiences (Table 2) led them to believe that they were not doing the right thing for themselves or their child.

### Superordinate Theme 4: An awareness of the child’s attachment and related difficulties

Awareness and understanding of their child’s difficulties is evidenced by all parents, who spoke coherently and empathically, reflecting a capacity to mentalise about the child’s issues, though for most, those issues involved alternative or additional explanations. Furthermore, parents struggled to accept that they had ‘done the best they could’ in their situation regarding their child’s attachment to them.

### Subordinate Theme 4.1: Advocating for my child (and other parents)

Some parents spoke about advocating for their child by trying to obtain help from schools and independent agencies, and by campaigning and community work. Some had gone further to advocate on behalf of other families in similar positions. While advocating was described as tiring and entailed great personal sacrifice financially, in loss or reduced employment, and time, their motivation came from wanting the best for their child, to increase public awareness and understanding of attachment difficulties, and to feel less lost in a system that had left them feeling dismissed and abandoned (though that feeling remained for many). Advocating gave parents more confidence about their child’s future by having appropriate support in place.

### Subordinate Theme 4.2: Seeking a further professional opinion

While HCP participants conceived that parents sought an alternative diagnosis because of their defensiveness to the attachment formulation, eight parents discussed how their instincts suggested that something else or more could explain their child’s presentation. Parents’ instincts were reinforced by family or other professionals which further motivated them to continue to push for an additional or alternative diagnosis. Parents reported that they wanted their gut feelings to be validated as reflected in the narrative of one parent who received an additional ADHD diagnosis for her son (Table 2). It is worth considering here that the delivery of attachment formulations was identified from some participant responses as having limited information and being inconsistent in approach, which might have influenced a parent’s decision to seek further professional opinion.

## Discussion

As the first study to explore the experiences of parents after receiving a clinical ‘formulation’ that their (biological) child had attachment-related difficulties, the findings bring to light the parent’s journey from shame to awareness. Parents’ profound distress, which ran centrally through the narratives, dissipated with the parents’ increasing awareness, consistent with research suggesting that higher levels of self-compassion help to downregulate shame-related distress and avoidance (Farr et al., 2021). Yet parents’ sense of failure as a parent persisted; whether resolution is achievable is unclear within the timeframe of this study. The attachment ‘label’ persists as a ‘label on self’.

The shame generated by the attachment-related ‘formulation’ was a painful inter-personal experience. Elison et al. (2006) outline four defence strategies used to manage shame: Avoidance, withdrawal, self-attack, and attack, the first three of which emerged in narratives. The consequences on parents’ mental and physical wellbeing, including self-harm and weight gain, may be viewed as resulting from maladaptive ways of managing parental distress. While parents’ reactions share features with those of parents informed of a new diagnosis for their child (e.g., feeling blamed and guilt; Smith-Young et al., 2020), the very ‘personal’ nature of the information combined with the fact that this is not a ‘clear-cut’ diagnosis adds to the sense of threat and confusion. The visceral, sensory and physiological reactions reported by parents are consistent with psychobiological shame responses (Dickerson et al., 2004). Critically, the ‘label’ of attachment-related difficulties offered no relief, clarity or any other positive dimensions, but was loaded with negative associations of feeling blamed and/or accused, confusion, stigma and isolation.

Given the relational nature of the issue (i.e., HCP-parent communication about a child-parent relationship), the parental journey can be altered by HCPs better understanding and showing greater sensitivity to the parental response. A future study could explore how HCPs decide on attachment-related difficulties as the best explanation for a child’s presenting problem and if/how specific models, training, supervision or service setting guide their decisions.

Furthermore, a key barrier to sense-making was considered by HCPs to be high defensiveness shown by parents. Avoidance and suppression defences are common in insecure adult attachment styles (Fraley & Shaver, 1997). However, a transactional explanation is also important to consider: the HCP’s own personal attachment style and negative feelings evoked by the parent’s perceived defensiveness might play a role in how helpful and trustworthy their information and service are perceived (e.g., Bucci et al., 2016). Parents placed high value on HCP characteristics that promote trust, which helped facilitate making sense. This is similar to the therapist’s role as a secure base to enable safe exploration of deeper issues emphasised in attachment-based psychotherapy (Farber et al., 1995).

Interestingly, when parents described being actively engaged with services and interventions, narratives around barriers and facilitators were imbued with relational language, perhaps signalling parents’ acknowledgement and motivation to repair. Facilitators included their desire to ‘reach’ their child and a sense of ‘connection’ that came from meeting others in a similar situation to them, while a prominent barrier was feeling ‘rejected’ and ‘abandoned’ by health services. From the HCP perspective, available interventions were sometimes perceived as too challenging to the parents’ psychological capacity for multiple reasons (defensiveness, discomfort during participation, and a lack of appropriateness or priority for the parent). Many parents also perceived the interventions offered as distressing, demanding or unnecessary, presenting a serious barrier to engagement, consistent with parenting intervention research (Axford et al., 2012; Hackworth et al., 2018).

The final stage of the journey describes the parents’ matured awareness of their child’s attachment difficulties and related difficulties. Taking on an advocating role is also described in studies of parents of children with other conditions (Smith-Young et al., 2020). However, most parents reported that, guided by their instinct, they also went on to seek additional or alternative professional opinions. While the HCPs in this study could view this as ‘defensive’, the seeking of additional diagnoses may be entirely appropriate given the high co-occurrence with other conditions that may be challenging to differentiate (Hudson et al., 2017).

### Limitations

The sample, predominantly white British women, is likely to reflect the experience of this demographic. Parents were volunteers, so may be more likely to have processed their experiences, engaged with relevant supports and/or felt particularly motivated to take part to express dissatisfaction with services. HCPs were interviewed based on their general experience of delivering attachment-related formulations, and recall may be easier for cases perceived as ‘difficult’ than not. Minimal information was (intentionally) collected around the child’s other issues and family history – which would have helped to contextualise parental responses (e.g., the seeking of alternative professional opinions). Finally, we were unable to collect information on the accuracy of the HCP’s formulation, what attachment models they drew on or how well they communicated their formulation to parents and how accurately parents processed this information.

### Clinical recommendations

Our findings suggest the need of an attachment-informed approach to prepare parents and HCPs, anticipating the challenges for both parties and often within an under-resourced context. Parents may be most receptive when HCPs work to normalise parents’ emotional reactions when receiving ‘hard to hear’ information and take an exploratory, supportive stance. HCP narratives in our study focused on parents’ perceived defensive strategies without reflecting on the possible activation of their own defences. However, activation of the attachment system can trigger defensive processes intra- and interpersonally, including transference and countertransference (McCluskey and O’Toole, 2019). Sensitive communication of a detailed, contextualised formulation that normalises attachment processes within a systemic framework is essential for optimising parental receptiveness by alleviating any felt shame and self-blame. The co-development with service users of information leaflets and other resources around attachment difficulties may help reduce stigma and enhance clarity to families. The grounded theory model presented in this paper could also be used to help normalise parents’ experiences along a common pathway.

Given the challenges with appropriate interventions from both parent and HCP perspectives, focused individual psychotherapeutic work and support of the parent are likely to be needed first, to address self-compassion and their own mental health, before these parents are psychologically able to engage in considering their child’s relational issues. Compassion-focused therapy was developed explicitly to support individuals with heightened shame and trauma histories. Additionally, interventions should consider building in a strong, high quality peer support component which would help normalise experience and reduce the sense of shame and stigma felt by parents.

## Conclusion

Under-resourcing and lack of appropriate attachment- and family-based interventions impact strongly on families for whom attachment-related difficulties are an identified issue; yet enhancing attachment and parental sensitivity continues to be a key mechanism for optimising child developmental and health outcomes (Bachmann et al., 2022). This is the case whether the attachment-related difficulties are considered to be primary or secondary to other neurodevelopmental or mental health conditions (e.g., Hudson et al., 2017; Kissgen et al., 2009). Our findings highlight how parents can be better supported into a space of awareness and understanding. The proposed model offers an empirically based and relatable common pathway to help normalise and validate parents’ experience. The study has also uncovered valuable insights relevant to all responsible for informing and improving patient care, including those influencing government policy, investment in services, mental health promotion, workforce development and supervision and service delivery. Future research directions could focus on reducing the identified barriers (and increasing the facilitators) to facilitate more positive outcomes for the child. The self-blame and shame parents experience when given a formulation or explanation of attachment-related difficulties is avoidable, if HCPs can support parents through this process in a sensitive and skilled ways.

## Supplemental Material

Supplemental Material - Navigating their child’s attachment-related difficulties: Parents’ journey from shame into awareness Click here for additional data file. Supplemental Material for Navigating their child’s attachment-related difficulties: Parents’ journey from shame into awareness by Chloe Crompton, Ming W Wan, Sarita Dewan and Anja Wittkowski in Clinical Child Psychology and Psychiatry.
