# Supplementary material for: Navigating their child’s attachment-related difficulties: Parents’ journey from shame into awareness
Source: Clin Child Psychol Psychiatry. 2022 Nov 9;28(4):1463–79. doi: 10.1177/13591045221135993 (PMC10540477; doi:10.1177/13591045221135993)
Supplement: Supplemental Material - Navigating their child’s attachment-related difficulties: Parents’ journey from shame into awareness [file sj-pdf-1-ccp-10.1177_13591045221135993.pdf]

Appendix A

INTERVIEW TOPIC GUIDE

(PARENTS)

Study Title: "Making sense of my child's emotional and relationship difficulties": A qualitative study.

Research Team: Chloe Crompton (Trainee Clinical Psychologist), Dr Anja Wittkowski (Senior Lecturer in Clinical Psychology, University of Manchester/Clinical Psychologist, Greater Manchester Mental Health Foundation Trust), Dr Ming Wai Wan (Lecturer in Developmental Psychology, University of Manchester), and Dr Sarita Dewan (Clinical Psychologist/Clinical Lead for Parent Infant Mental Health, Pennine Care NHS Foundation Trust)

What follows is a guide to the topics which are likely to be covered in the interviews with parents to explore their experiences of receiving their child's attachment-related formulation. The order and content of the questions may vary do to the open-ended questions and discussions that may follow as the interview develops. The questions will be influenced by on-going analysis after each interview.

Request a brief timeline through service from point of referral through to learning final outcome of attachment difficulties (i.e. number of appointments attended, reason for appointment etc.)

- What is your understanding of what you've been told about your child from (*relevant NHS service name*)?
  Additional probes:
    - What is your child's behaviour like?
    - Does what (*relevant NHS service name*) fit for what you thought about your child before being seen?

- Please tell me about how you learned about the referral for your child to (*relevant NHS service name*)?
  Additional probes:
    - Who made the referral?
    - How did the referral happen?
    - Why did the referral happen?

- What were your initial thoughts about the referral?
- What was your understanding of how the referral came about? (NOV 2020) How was it communicated to you?
- (NOV 2020) What was your understanding of CAMHS (i.e. being provided with information about the process/appointments)?

- Please tell me about what was going on for you when you were seen by (*relevant NHS service name*)?

  Additional probes:
  - How did it influence how you felt about yourself?
  - How did it make you think about your parenting?
  - How did it make you feel about your relationship with your child?
  - How did you view yourself/your child?
  - Was there anything which was helpful/unhelpful (service or personal)? (NOV 2020: note – characteristics/approach of the clinician, personal characteristics)

- (NOV 2020) Please tell me how you felt when learning about/receiving the opinion of your child's attachment difficulties?

  Additional probes:
  - How did you manage/still manage those feelings?
  - What could have been done to help you manage those feelings? What could the service have done? What could have the clinician have done? What else could you have done differently?
  - Have these feelings changed? If so, what has helped?

- Please tell me how have you made sense of what has been going on for your child?

  Additional probes:
  - What was your initial reaction?
  - What went through your mind?
  - How did this fit with your understanding of the referral?
  - How has it altered the way you think about (*child's name*)?
  - How has it altered the way you think about yourself?
  - (Jan 2021) How did you feel others perceived you as a parent?
  - What were some of the helpful things to support your understanding?
  - (NOV 2020) How did completing your own research support your understanding? Why did this feel important to do?
  - What were some of the unhelpful things which prevented you making sense of this?
  - What do you think could have been more helpful to support your understanding?
  - (NOV 2020) Have you talked openly about your child's attachment difficulties with friends/family/within your community? What has helped you to do this? What barriers have you met by doing this?
  - (NOV 2020) What do you think is needed for more people to understand attachment difficulties?

- Please tell me about any other support you received, either from other parents or professionals?

  Additional probes:
  - How did you feel about the support?
  - What went through your mind?
  - How did you feel about continuing to engage with (*relevant NHS service name*)?
  - (NOV 2020) How easy was it to access the support and use the strategies you were given?
  - (NOV 2020) How helpful was the support for enhancing your understanding?
  - Was there anything you found helpful/unhelpful (service or personal)?
  - (NOV 2020) What is it that has kept you engaging with *(support)*?
  - (NOV 2020) What is it about you/your personality that helps you to keep going?
  - (NOV 2020) How is your relationship with your partner/family and how have they supported you and your child?
  - (NOV 2020) What was your partners understanding? Was it the same or different to yours?
  - (NOV 2020) How have you found the support from school/your employer?

- How would you describe your experience of accessing (*relevant NHS service name*)?

  Additional probes:
  - How is your understanding of your child after going through this process?
  - How did it feel going through it as (*child's name*) mum/dad?

Additional questions to consider:

- Have you ever experienced mental health difficulties? (current or past)
  - What type of support/input did you receive (medication, counselling, online, peer, psychological therapy)?
- Do you know anyone else who has been through a similar process?
  - Do you find you compare yourself to them?
- Can you tell me about your own experiences of when you were younger from your parents?
  - How do you find your parenting in comparison to your experience of your own parents?
  - (NOV 2020) Has going through this process led you to reflect on your own childhood experiences of being parenting/relationships with your parents? Or What has helped you to reflect on your own childhood experiences/relationships with your parents?
  - (NOV 2020) How have your own childhood experiences impacted on your parenting style?
  - (JAN 2020) Have you ever discussed or thought about your own attachment patterns? How has this been helpful?

Appendix B

INTERVIEW TOPIC GUIDE

(CLINICIANS)

Study Title: "Making sense of my child's emotional and relationship difficulties": A qualitative study.

Research Team: Chloe Crompton (Trainee Clinical Psychologist), Dr Anja Wittkowski (Senior Lecturer in Clinical Psychology, University of Manchester/Clinical Psychologist, Greater Manchester Mental Health Foundation Trust), Dr Ming Wai Wan (Lecturer in Developmental Psychology, University of Manchester), and Dr Sarita Dewan (Clinical Psychologist/Clinical Lead for Parent Infant Mental Health, Pennine Care NHS Foundation Trust)

What follows is a guide to the broader topics which are likely to be covered in the interviews with clinicians to explore their experiences of delivering an attachment-related formulation to a parent. The order and content of the questions may vary and discussions that may follow as the interview develops. The questions will be influenced by on-going analysis after each interview.

Topic 1: How the service receives referrals for children with considered attachment difficulties.

- Parents awareness of referral.
- Support offered from services.

Topic 2: Clinicians experience of delivering an attachment-related formulation.

- Wording or terminology used; (NOV 2020) including any phrases which are avoided, how are attachment-related formulations communicated to parents, how does this impact parent response/reaction/feelings, how much education is given around the term.
- Clinician's relationship with parent/child.
- Setting the scene for a parent.
- Clinician's feelings before, during and after (NOV 2020: less prompting required).
- Personal experiences impacting on approach (NOV 2020: less prompting required).
- Approaches for adoptive and birth parents (NOV 2020: less prompting required).
- (NOV 2020) Pressures of services (note: limited resources, financial strain, working to meet targets).
- (NOV 2020) How are parents own attachment patterns explored? How is insight assessed before delivering the formulation?

Topic 3: Clinicians perception of parent receiving attachment-related formulation.

- (NOV 2020) When it has been received well and not received well.
- Parents thoughts about referral.
- (NOV 2020) The strategies/experiences which impact how ready parent is to hear about their child attachment difficulties (note: own secure base, being in tune with own emotions, processed own traumas, awareness of own attachment patterns)
- Parent characteristics (i.e. confidence, resilience, NOV 2020; reflective capacity, defence mechanisms) and impact on sense-making process.
- Parents reactions (NOV 2020) and what can services do to help.
- (NOV 2020) Characteristics/approach of the clinician (i.e. consistent, empathic, playful, pacing - note: how aware is the parent that they need the clinician to be this way).
- Meanings attached to formulation (NOV 2020) on an individual, family service and societal level.
- (NOV 2020) Parents understanding of attachment pre-referral and post-referral.
- (NOV 2020) Googling attachment (note: impact of sense making due to varying definitions/types of attachment).
- Parents thoughts about themselves or thoughts about their child before and after.
- (JAN 2021) Parents perceptions of how others think, feel, act towards them.
- Strategies and support that parent receives and how easy it is for a parent to implement those strategies or access/engage with support (i.e. attending groups – what keeps them coming back/or avoid?)

Appendix C

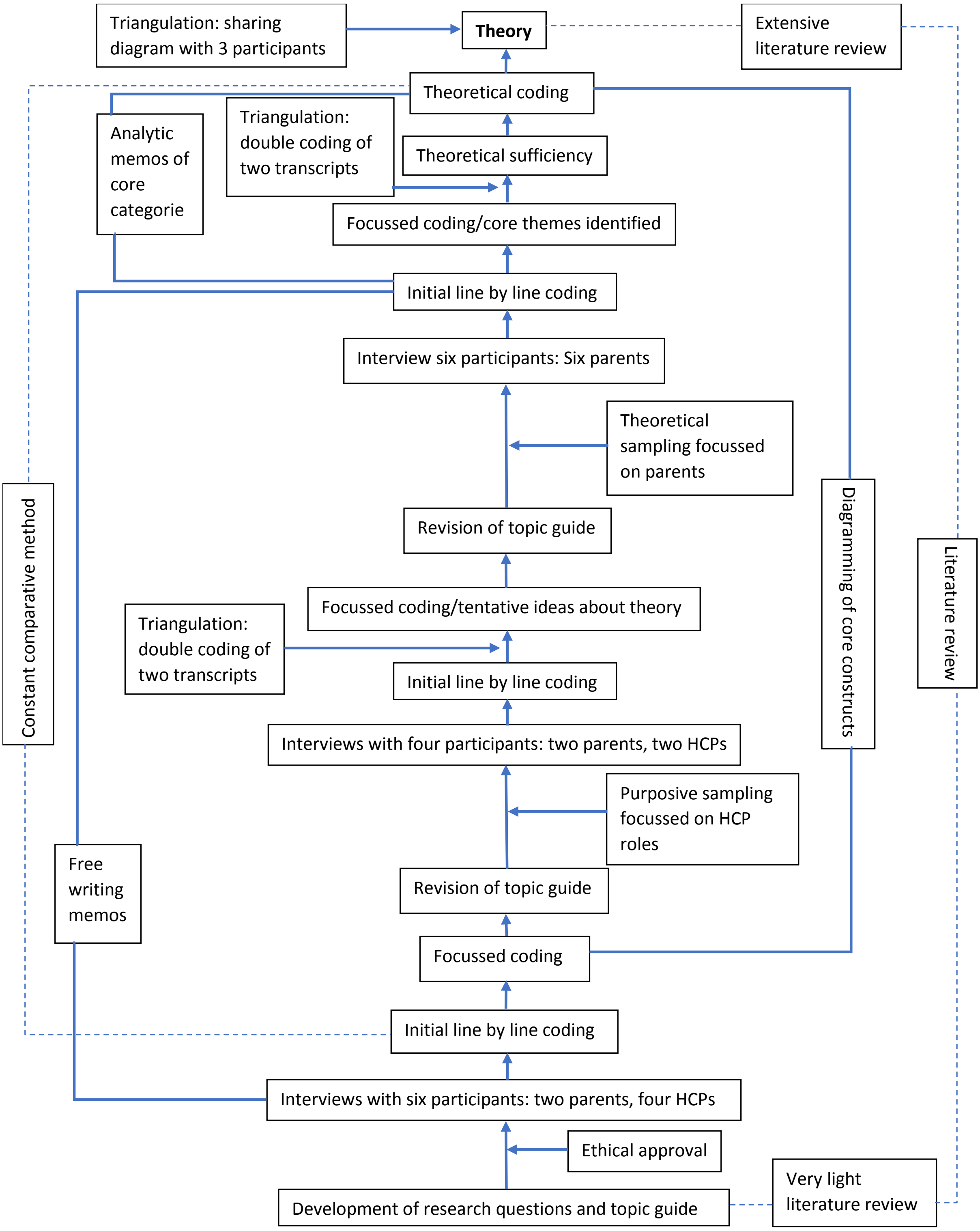

Flow diagram to demonstrate this study's grounded theory methodology
